# Systematic Analysis of the Expression and Prognostic Significance of P4HA1 in Pancreatic Cancer and Construction of a lncRNA-miRNA-P4HA1 Regulatory Axis

**DOI:** 10.1155/2020/8877334

**Published:** 2020-12-19

**Authors:** Zhili Hu, Fang Song, Yangzhi Hu, Tianyou Liao

**Affiliations:** ^1^Department of Gastrointestinal Surgery, Liuzhou People's Hospital, Liuzhou, Guangxi Province 545006, China; ^2^Department of Gastrointestinal Surgery, Affiliated Hospital of Xiangnan University, Chenzhou, Hunan Province 423000, China; ^3^Department of Gastroenterology, Zaoyang First People's Hospital, Zaoyang, Xiangyang, Hubei Province 441200, China; ^4^Department of Gastrointestinal Surgery, Shunde Hospital, Southern Medical University (The First People's Hospital of Shunde Foshan), Shunde, Foshan, Guangdong Province, 528300, China

## Abstract

**Objectives:**

Prolyl 4-hydroxylase subunit alpha 1 (P4HA1) plays a crucial role in modulating extracellular matrix component and promoting tumor progression by changing tumor adhesion, migration, and other biological behaviors in some cancers. However, its expression pattern, biological function, and underlying mechanism in pancreatic cancer remain largely unclear.

**Materials and Methods:**

In this study, a set of bioinformatics tools were used to analyze the expression of P4HA1 and its prognostic value in pancreatic cancer. In addition, the mechanism through which P4HA1 promotes the progression of pancreatic cancer was explored by constructing a competing endogenous RNA (ceRNA) regulatory axis.

**Results:**

It was found that the mRNA and protein expression of P4HA1 was significantly higher in pancreatic cancer tissues than in normal tissues. Its high P4HA1 expression correlated with poor clinicopathological features (T stage: *P* = 0.0078; N stage: *P* = 0.0124; TNM stage: *P* = 0.0013; pathological grade: *P* = 0.0108) and poor prognosis [OS: HR = 1, 95% CI (1-1.01), *P* = 0.00028; DSS: HR = 1, 95% CI (1-1.01), *P* = 0.00049; PFI: HR = 1.01, 95% CI (1.01-1.02), *P* = 0.0057; and DFI: HR = 1, 95% CI (1-1.01), *P* = 0.0034]. The LINC01503/miR-335-5p/P4HA1 axis might mediate the effects of P4HA1 in promoting the progression on pancreatic cancer.

**Conclusions:**

Collectively, our findings suggest that high expression of P4HA1 may be used as a promising prognostic biomarker and could be considered for the development of a novel therapeutic strategy for pancreatic cancer in the future.

## 1. Introduction

Pancreatic cancer is one of the most malignant tumors with the worst prognosis in the world, and it ranks seventh among the cancer-related deaths in the world and fourth amid all the cancer-related mortalities in the United States; annually, about 432,000 patients die of pancreatic cancer every year^1^. In the recent years, although some progress has been made in the field of combined and precision therapy for pancreatic cancer. However, overall prognosis remains very poor with a 5-year survival rate of less than 6%^2^. This makes the cancer one of the deadliest malignant tumors worldwide.

Prolyl 4-hydroxylase subunit *α*1 (P4HA1), located at 10q22.1, is the active catalytic subunit of prolyl 4-hydroxylase (P4H). P4HA1 is responsible for newly synthesized collagen chains and plays a significant role in the formation and stability of the triple helix region of newly synthesized collagen chains. Additionally, it is the key catalytic subunit in regulating collagen biosynthesis^3^. Notably, recent studies found that P4HA1 not only could regulate extracellular collagen synthesis and secretion but also were closely related to the progression of some tumors, such as breast cancer^4^, prostate cancer^5^, and glioma^6^, ^7^ by changing tumor adhesion, migration, and other biological behaviors. However, the association between P4HA1 and pancreatic cancer as well as its role in the progression of the tumor was not clearly shown.

Human genome-wide and transcriptome research data shows that protein-coding genes account for only a tiny proportion of the genome (1-2%), while most of the genes in the genome are transcribed; they encode large amount of nonprotein coding RNA (ncRNA) much more than we expected^8^, ^9^. Interestingly, at least 80% of the ncRNA in the human genome is bioactive as opposed to the previous belief that it was simply “junk product” 10. Based on the length of the transcript, ncRNA is mainly divided into two categories: short-stranded noncoding RNA, such as microRNA (miRNA) (<200 bp), and long noncoding RNA (lncRNA) (>200 bp). In 2011, Salmena et al. 11 proposed a competing endogenous RNA (ceRNA) theory. They postulated that the 3′UTR of lncRNA and mRNA are both rich in miRNA recognition elements (MRE). Moreover, lncRNA could adsorb miRNA through MRE and competitively bind miRNA to inhibit its activity. This happened in order to partially remove the inhibitory effect of miRNA on its target genes and upregulate their expression. Therefore, miRNA and lncRNA play a molecular regulatory role in many processes of gene expression, participate in the pathophysiological process of tumors, and are closely related to the occurrence and progression of cancer^12^. Moreover, the mechanism of the ceRNA of P4HA1 affecting the malignant progression of pancreatic cancer has not been reported.

Therefore, the expression and clinical value of P4HA1 in pancreatic cancer were comprehensively analyzed in this study. Additionally, a ceRNA regulatory axis was constructed to explore the potential mechanism of P4HA1 in the progression of pancreatic cancer.

## 2. Materials and Method

### 2.1. Pan-Cancer Analysis of P4HA1 in the Oncomine Database

Oncomine (http://www.oncomine.org/resource/login.html) is an open-access cancer microarray database and an integrated data mining platform that facilitates the discovery of valuable clues from genome-wide expression analysis. So far, the database has 19 cancer types and contains 92 datasets^13^. In this study, Oncomine was used to compare the 19 cancer samples with normal samples and identify the expression of P4HA1 in various tumors. At the same time, the expression levels of P4HA1 mRNA (log2 transformed) in pancreatic cancer and normal tissues were examined in order to determine the transcription level of P4HA1 in the disease (*P* < 0.001, ∣fold change | >2, and the top 10% of the gene ranking as the threshold condition).

### 2.2. Pan-Cancer Analysis of P4HA1 in the Sangerbox Database

The Sangerbox database (http://sangerbox.com/Tool) is a comprehensive Chinese bioinformatics analysis platform. The pan-cancer analysis tool of this database was used to explore the expression of P4HA1 in pancreatic cancer and normal samples (^∗^*P* < 0.05, ^∗∗^*P* < 0.01, and ^∗∗∗^*P* < 0.001). In addition, this analysis platform was also used to investigate the effect of P4HA1 on the survival and prognosis of pancreatic cancer. This included an analysis of overall survival (OS) and disease-specific survival (DSS). A log-rank *P* value < 0.05 was considered to be statistically significant.

### 2.3. Expression, Prognosis, Coexpression, and Correlation Analysis of P4HA1 in the GEPIA Database

Gene Expression Profiling and Interactive Analysis (GEPIA) (http://gepia.cancer-pku.cn/index.html) is an interactive platform including 9,736 tumors and 8,587 normal samples from The Cancer Genome Atlas (TCGA) and Genotype-Tissue Expression (GTEx) projects. It helps in the analysis of RNA sequencing expression data^14^. GEPIA provides customizable functions, such as tumor/normal differential expression analysis, patient survival analysis, similar gene detection, and correlation analysis. The database uses the log-rank (Mantel-Cox) test in the prognosis of 33 different types of cancer based on gene expression. Moreover, the box plot was used to display the ordinate of gene expression in tumor/normal differential expression analysis. The expression data was converted by log2 (TPM+1), ∣log2 FC | >1, and *P* value < 0.05 was set as the truncation criterion. Finally, gene expression correlation analysis of the given TCGA expression dataset was used to determine the correlation coefficient using the Pearson method.

### 2.4. The Expression of P4HA1 in the HPA Database

The Human Protein Atlas (HPA) (http://www.proteinatlas.org/) provides tissue and cellular distribution information for 26,000 types of human proteins^15^. In this database, the researchers used highly specific antibodies and immuno-detection techniques (immunoblotting, immunofluorescence, and immunohistochemistry) to identify in detail the expression of each protein in 64 cell lines, 48 normal human tissues, and 20 tumor tissues. The immunohistochemical staining data from the Tissue and Pathology section of the HPA was used to detect the expression level of the P4HA1 protein in human pancreatic cancer and normal pancreatic tissues.

### 2.5. TCGA-PAAD Data Download and Data Analysis in UCSC Xena

The University of California Santa Cruz (UCSC) Xena browser (https://xenabrowser.net/) was used to obtain detailed information on mRNA expression of genes. In addition, the browser was employed to source clinical information on pancreatic adenocarcinoma (PAAD) patients in the TCGA cohort (Project ID: TCGA-PAAD)^16^, ^17^. Moreover, the relationship between the expression of P4HA1 and clinicopathological parameters (gender, T stage, N stage, M stage, TNM stage, and pathological grade) in pancreatic cancer was analyzed using the TCGA-PAAD data downloaded from Xena.

### 2.6. Screening for Genes Coexpressed with P4HA1

Screening for genes coexpressed with P4HA1 was done using the GEPIA and UALCAN databases. Afterwards, the same coexpression genes in the two databases were screened and a Venn diagram was drawn using the intersection method.

The UALCAN database is a user-friendly and interactive network resource, which provides a convenient way of publicly obtaining cancer transcriptome data from TCGA^18^. In this study, the database was used to find genes coexpressed with P4HA1 in PAAD. Additionally, expression of some genes/miRNAs was analyzed.

### 2.7. GO Analysis and KEGG Pathway Analysis of Coexpressed Genes

Enrichr (http://amp.pharm.mssm.edu/Enrichr/) is a comprehensive gene set enrichment analysis web server^19^, ^20^. It was used to perform Gene Ontology (GO) functional annotation and Kyoto Encyclopedia of Genes and Genomes (KEGG) pathway enrichment analysis on P4HA1 and its coexpressed genes. The top 10 rich enrichment projects and paths were displayed on the webpage and could be downloaded directly.

### 2.8. PPI Analysis of Coexpressed Genes and Hub Genes Were Identified

In this study, the String database (http://string-db.org)^21^ and Cytoscape (version 3.4.0) software^22^ were used to construct a protein-protein interaction (PPI) network of coexpressed genes. The Hub gene identifying plug-in cytoHubba (version 0.1) is a Cytoscape application that uses several topological algorithms to predict and identify important nodes and subnets in a given network. Moreover, Molecular Complex Detection (MCODE) (version 1.6.1) is an application plug-in for Cytoscape that is used to cluster a given network according to topology, in order to find closely connected areas. The cytoHubba and MCODE were used to identify the most important genes and module in the PPI network, respectively.

### 2.9. Expression, Correlation, and Pathway Enrichment Analysis of P4HA1 and Top 15 Hub Genes

The TCGA data downloaded from the Xena browser was used to show the expression patterns of P4HA1 and obtained Hub genes in a heat map. Additionally, correlation analysis and KEGG enrichment analyses were performed on these genes. The results of gene enrichment analysis could intuitively show which genes were enriched in each pathway in a circos diagram.

### 2.10. Analysis of Three Hub Genes Directly Interacting with P4HA1

The String database was used to construct a PPI network between P4HA1 and its top 15 coexpressed Hub genes. The three Hub genes directly interacting with P4HA1 were screened out, and a PPI network was performed. Afterwards, the three screened Hub genes were analyzed for expression and correlation using the GEPIA database.

### 2.11. NetworkAnalyst Database

The NetworkAnalyst (http://www.networkanalyst.ca/faces/home.xhtml) is an online website for comprehensive gene expression analysis, meta-analysis, and biological network regulation analysis^23^, ^24^. To further explore the mechanism of P4HA1 underlying the prognosis of pancreatic cancer, the Gene-miRNA Interactome module of the NetworkAnalyst database was used to predict the upstream miRNAs that could regulate P4HA1. The predicted data originated from comprehensive experimental verifications collected in TarBase and miRTarBase miRNA-Gene interaction information.

### 2.12. Predictive miRNA Expression, Correlation, and Prognostic Analysis

The UALCAN database was used to compare the expression of 5 miRNAs between normal and pancreatic cancer tissues. Moreover, the LinkedOmics database (http://www.linkedomics.org/login.php) 25 was used to analyze the correlation between 5 miRNAs and the expression level of P4HA1 in pancreatic cancer. It is well known that a negative regulatory relationship exists between miRNA and targeted mRNA. Therefore, miRNA that negatively correlated to P4HA1 expression was considered to have good research prospects.

The Kaplan-Meier Plotter (http://kmplot.com/analysis/) is an online database established from gene expression data and survival information of patients with cancer^26^. Using TCGA data, this database was utilized to evaluate the prognostic value of the predicted miRNAs in PAAD. The miRNA name was keyed into the database, after which its Kaplan-Meier survival plots, hazard ratio (HR), 95% confidence interval (CI), and log-rank *P* values were displayed directly on the web page.

### 2.13. Analysis of Upstream lncRNA Targeted to miRNA

Here, the StarBase (http://starbase.sysu.edu.cn/index.php)^27^, ^28^ and the miRNet (https://www.mirnet.ca/) databases were introduced to predict lncRNAs that could bind miR-335-5p. Subsequently, the lncRNAs that predicted from the StarBase were intersected with the predicted from miRNet was considered to be the most potential regulatory lncRNA for miR-335-5p. According to the regulatory pattern of ceRNA, there is a negative correlation between the expression of lncRNA and miRNA and a positive correlation with the expression of target genes. Based on this feature, the study further screened for the most likely regulatory lncRNA targeted to miR-335-5p by correlation analysis. The StarBase was used to analyze the correlation between lncRNA-miRNA and lncRNA-P4HA1. *R* > ∣0.1∣ and *P* < 0.05 were set as the critical criteria for identifying important miRNA-lncRNA and P4HA1-lncRNA pairs.

### 2.14. A ceRNA Regulation Axis and Brief Mechanism Diagram of P4HA1

The study combined the coexpressed Hub genes directly interacting with P4HA1 obtained from above process, upstream miRNA for P4HA1 and upstream lncRNA for miRNA to construct a ceRNA regulatory lncRNA-miRNA-P4HA1 network diagram. Additionally, previous relevant reports from the literature were combined to draw a brief model diagram of the mechanism by which P4HA1 promotes the progression in pancreatic cancer.

### 2.15. Statistical Analysis

By default, all statistical analyses were performed as described in the web resources. Briefly, the *t*-test was performed to compare the expression of P4HA1 mRNA in the Oncomine database. The *t*-test was also used to perform comparative analysis of P4HA1 expression among different subgroups for each clinicopathological feature. The log-rank test was used to calculate the *P* value for survival data in the Sangerbox, GEPIA, and Kaplan-Meier Plotter databases. Furthermore, the Fisher's exact test was used to measure gene enrichment in annotated items of the Enrichr annotation system. Finally, linear correlation between mRNA-mRNA, miRNA-mRNA, and lncRNA-miRNA was measured using Pearson's correlation coefficient in the GEPIA, LinkedOmics, and StarBase databases. *P* < 0.05 was considered statistically significant (^∗^*P* < 0.05, ^∗∗^*P* < 0.01, and ^∗∗∗^*P* < 0.001).

## 3. Results

### 3.1. The Expression and Prognosis of P4HA1 in Pan-Cancer

**Figure 1:**
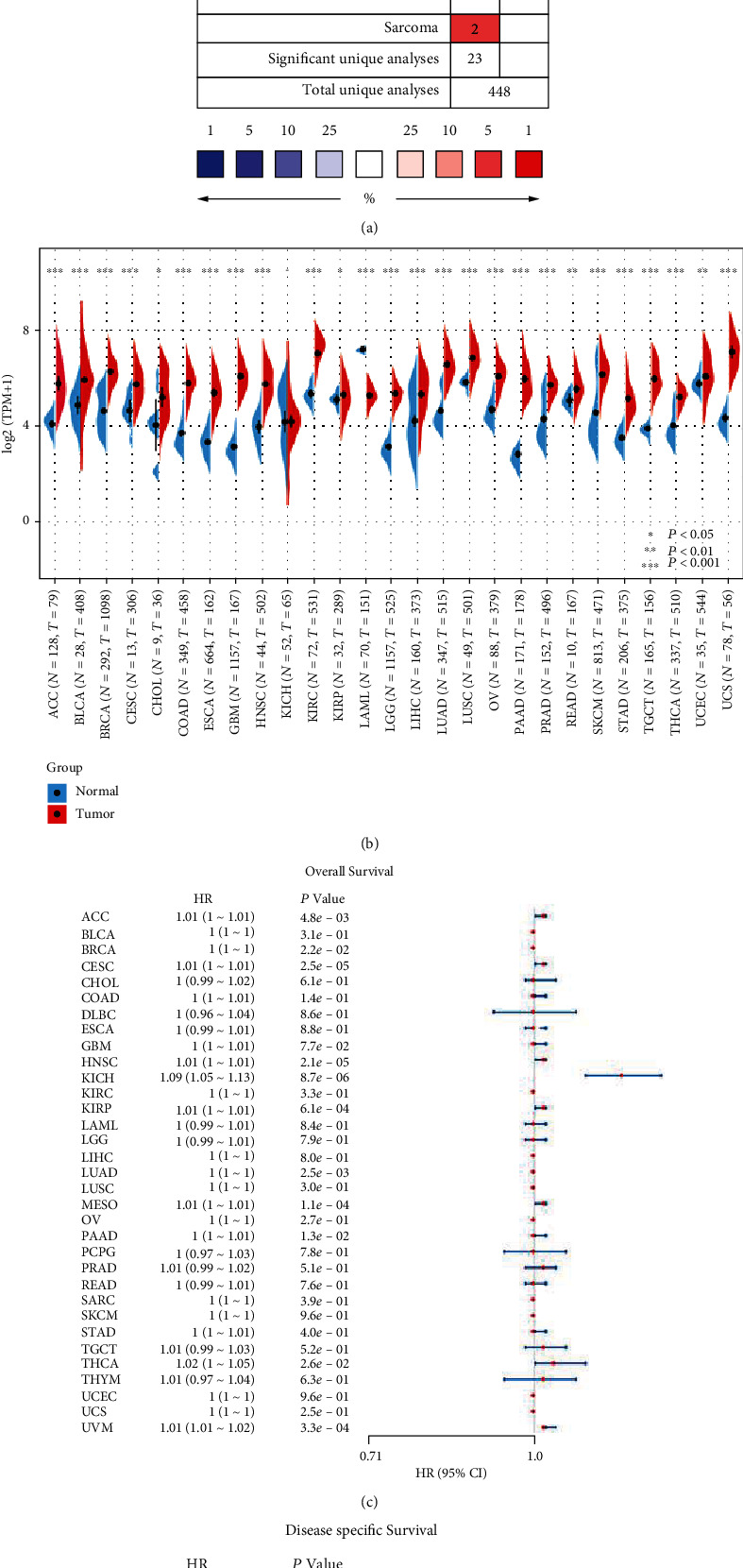
Expression pattern and prognostic significance of P4HA1 in human pan-cancer. (a) Expression of P4HA1 in various cancers by Oncomine analysis of cancer samples versus normal samples. (b) Comparison of P4HA1 expression between pan-cancer samples and paired normal samples based on TCGA and GETx data. (c, d) The effect of P4HA1 expression on the overall survival (OS) and disease-specific survival (DSS) of pan-cancer based on TCGA data, respectively.

The overall expression profile of P4HA1 in various cancers was first analyzed using the Oncomine database by making comparisons between cancer and normal tissue samples. P4HA1 was markedly overexpressed in most tumors, especially in kidney and pancreatic cancers (Figure 1(a)). Afterwards, TCGA and GTEx data were used to analyze the expression of P4HA1 in pan-cancer, in order to further quantitatively verify its expression in various tumors.  Figure 1(b) shows that there was a significant difference in the expression of P4HA1 between normal and tumor samples of most tissues except for the Kidney Chromophobe (KICH). Furthermore, P4HA1 was overexpressed in most tumors. Data from TCGA was also used to analyze the impact of high P4HA1 expression on the OS (Figure 1(c)) and DSS (Figure 1(d)) of patients. This was done in order to understand further the impact of P4HA1 on the prognosis of survival from each tumor. Figures 1(c) and 1(d) show that the expression of P4HA1 affects the OS and DSS of patients with PAAD.

In the above preliminary investigation, P4HA1 was significantly overexpressed in pancreatic cancer. Additionally, it was related to poor prognosis of survival in patients with PAAD warranting further study.

### 3.2. Expression of P4HA1 in Pancreatic Cancer

**Figure 2:**
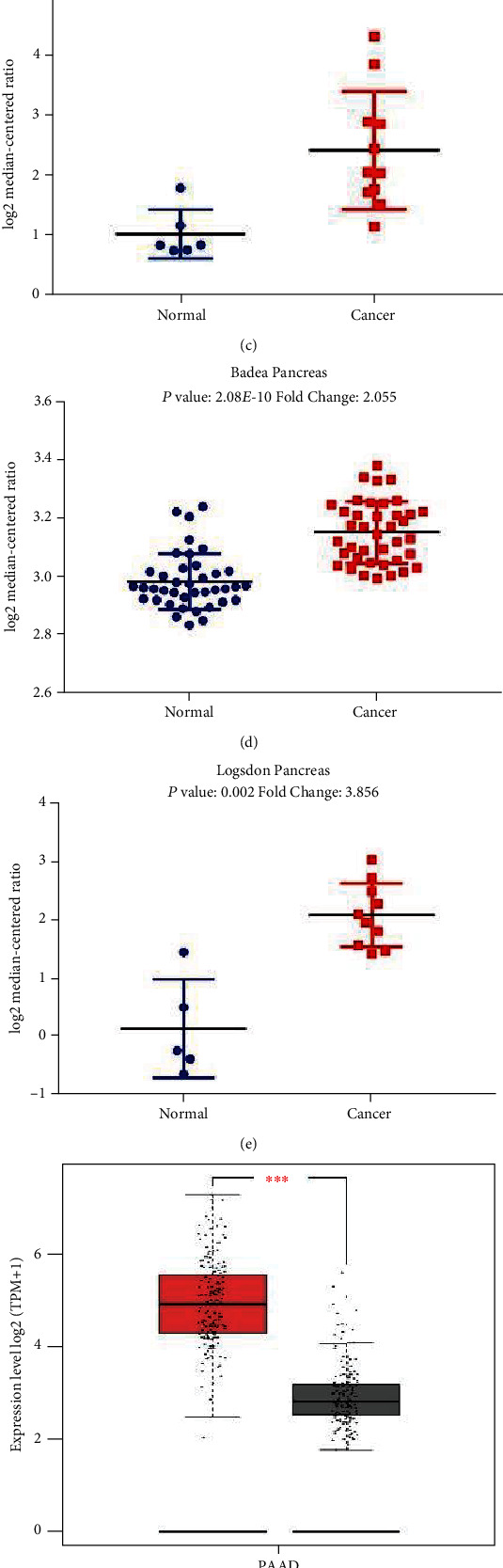
P4HA1 expression in normal pancreatic tissues and pancreatic adenocarcinoma (PAAD) tissues. (a–e) P4HA1 mRNA expression in four pancreatic cancer studies based on analysis of Oncomine database. (f) Expression of P4HA1 in PAAD tissues and normal pancreatic tissues based on TCGA and GETx datasets using the GEPIA database. Error bars represent SD. ^∗∗∗^*P* < 0.001. (g, h) Immunohistochemical staining of P4HA1 protein, respectively, showed the protein expression levels in normal pancreatic tissues and pancreatic cancer tissues based on data from the HPA database.

The analysis of tumor vs. normal samples in different datasets from Oncomine showed that P4HA1 was significantly overexpressed in multiple pancreatic cancer datasets (Figures 2(a)–2(e)). The GEPIA database was also used to further confirm that P4HA1 was significantly highly expressed in the TCGA-PAAD dataset (Figure 2(f)). In addition, the HPA database showed that immunohistochemical staining of cancer tissues was deeper than that of normal pancreatic tissues. This suggests that there was remarkable expression of the P4HA1 protein in pancreatic cancer tissues.

**Figure 3:**
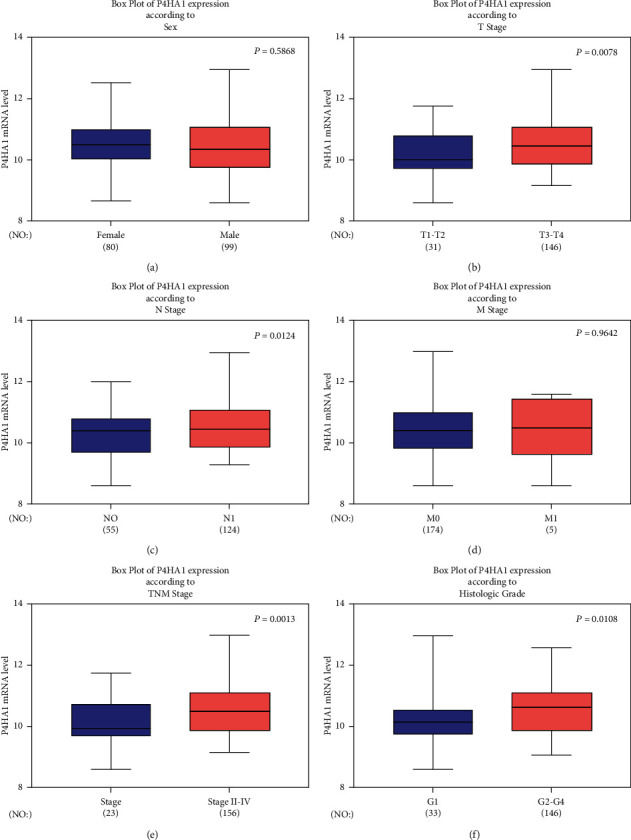
Association between P4HA1 expression and various clinical-pathological parameters in pancreatic cancer based on data from the TCGA. (a) A boxplot showing expression of P4HA1 based on gender. (b) A boxplot showing P4HA1 expression according to T stage. (c) A boxplot showing expression of P4HA1 according to nodal status. (d) A boxplot showing expression of P4HA1 according to metastatic status. (e) A boxplot showing expression of P4HA1 according to TNM stage. (f) A boxplot showing expression of P4HA1 according to histological grade.

The study further explored the association between P4HA1 expression and different clinical-pathological features in patients with PAAD (Figure 3). The results showed that there was no significant difference in the expression of P4HA1 between male and female patients as shown in Figure 3(a) (*P* = 0.5868). Additionally, there was no significant difference between the positive and the negative metastasis groups as highlighted in Figure 3(d) (*P* = 0.9642). In terms of T stage (T1-2 vs. T3-4), N stage (N- vs. N+), TNM stage (I vs. II-III), and pathological grade (G1 vs. G2-4), expression of P4HA1 was significantly high in the subgroups with high stage/grade (T stage: *P* = 0.0078; N stage: *P* = 0.0124; TNM stage: *P* = 0.0013; pathological grade: *P* = 0.0108). Therefore, these results indicate that P4HA1 expression was associated with unfavorable clinicopathological characteristics in patients with pancreatic cancer and might play a detrimental role in pancreatic cancer.

All in all, the findings show that P4HA1 is a reliable and promising biomarker of pancreatic cancer.

### 3.3. Prognostic Significance of P4HA1 Gene in Pancreatic Cancer

**Figure 4:**
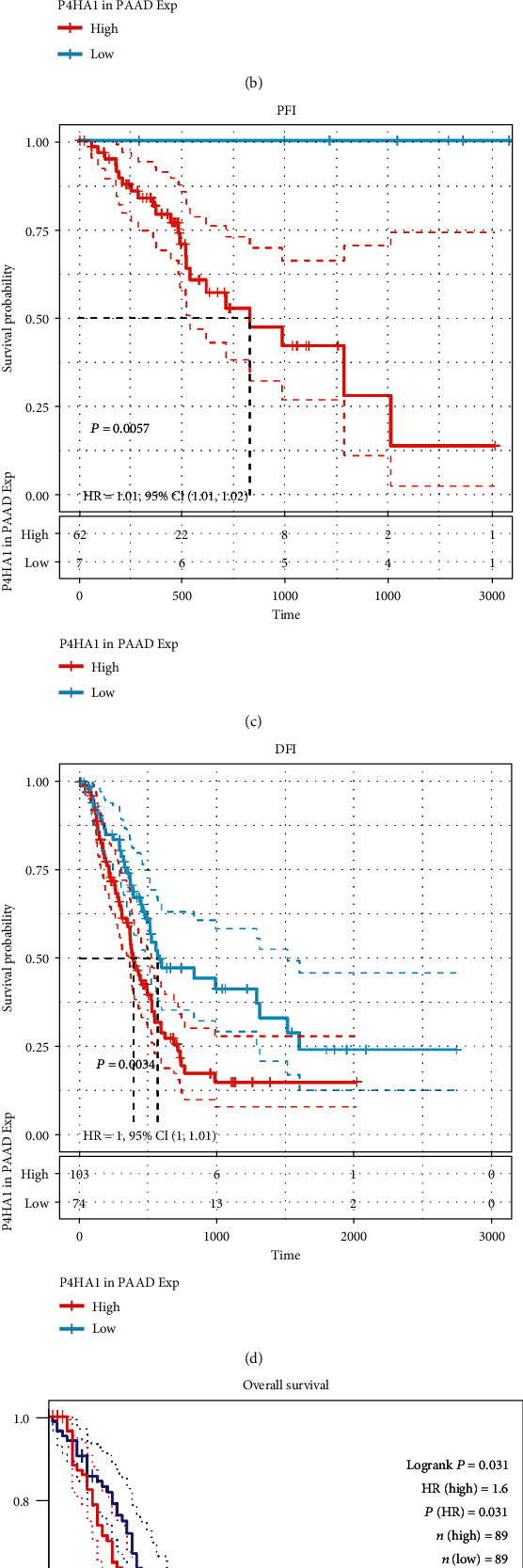
Prognostic value of P4HA1 in pancreatic cancer. (a–d) High expression of P4HA1 correlated with poor OS, DSS, PFI, and DFI, respectively, in PAAD (TCGA database). (e, f) High expression of P4HA1 correlated with poor OS and DFS of PAAD (GEPIA database).

Three online databases including Sangerbox and GEPIA were used to further evaluate the prognostic value of P4HA1 in human pancreatic cancer (Figure 4). Patients with high P4HA1 expression showed poor OS [OS: HR = 1, 95% CI (1-1.01), *P* = 0.00028], DSS [DSS: HR = 1, 95% CI (1-1.01), *P* = 0.00049], progress-free interval (PFI) [PFI: HR = 1.01, 95% CI(1.01-1.02), *P* = 0.0057], and disease-free interval (DFI) [DFI: HR = 1, 95% CI (1-1.01), *P* = 0.0034] as shown in Figures 4(a)–4(d) (SangerBox). Results of the GEPIA database analysis similarly indicated that the high expression of P4HA1 indicated poor prognosis in pancreatic cancer patients [OS: HR = 1.6, *P* = 0.031; DFS: HR = 1.7, *P* = 0.014] (Figures 4(e) and 4(f)). All these findings showed that P4HA1 was associated with poor prognosis in patients with pancreatic cancer. It could therefore be used as a predictor of poor prognosis in the disease.

### 3.4. GO and KEGG Pathway Analysis of Genes Coexpressed with P4HA1

**Figure 5:**
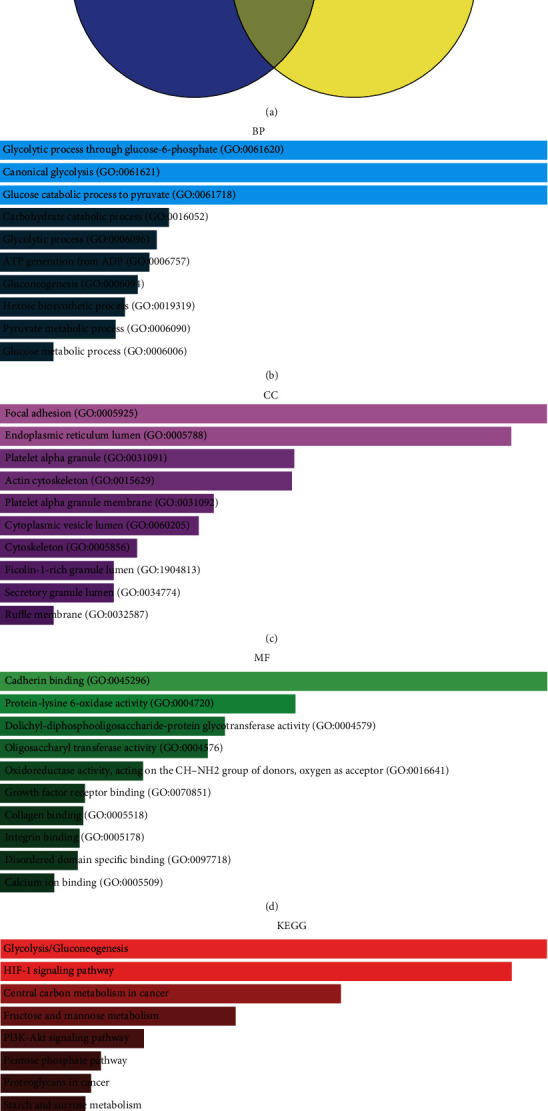
GO and KEGG pathway enrichment analyses of coexpressed genes. (a) A Venn diagram showing the intersection of coexpressed genes between UALCAN database and GEPIA database. A total of 139 coexpressed genes of P4HA1 were identified. (b–d) GO analysis revealed that the 139 coexpressed genes of P4HA1 were represented in biological process (BP), cellular component (CC), and molecular function (MF), respectively. (e) KEGG pathway enrichment analysis for the 139 coexpressed genes.

Identifying genes coexpressed with P4HA1 would help to better understand the potential function of the protein in pancreatic cancer. Therefore, the UALCAN and GEPIA databases were used to identify these coexpressed genes, as shown in Figure 5(a). A total of 139 coexpressed genes were predicted by both databases. Afterwards, the GO enrichment (Figures 5(b)–5(d)) and KEGG pathway enrichment analyses were performed on the genes (Figure 5(e)). Additionally, the Enrichr database was used to perform the KEGG pathway enrichment and functional annotation analyses including three types of GO analysis: biological processes (BP), cellular components (CC), and molecular functions (MF).

The GO functional enrichment of these coexpressed genes in the BP category included the following: the glycolytic process through glucose-6-phosphate (GO:0061620), canonical glycolysis (GO:0061621), glucose catabolic process to pyruvate (GO:0061718), glycolytic process (GO:0006096), and generation of ATP from ADP (GO:0006757). In the CC category, it included focal adhesion (GO:0005925), endoplasmic reticulum lumen (GO:0005788), platelet alpha granule (GO:0031091), actin cytoskeleton (GO:0015629), and platelet alpha granule membrane (GO:0031092). Finally, the MF group included the following: cadherin binding (GO:0045296), protein-lysine 6-oxidase activity (GO:0004720), dolichyl-diphosphooligosaccharide-protein glycotransferase activity (GO:0004579), oligosaccharyl transferase activity (GO:0004576), and oxidoreductase activity, acting on the CH-NH2 group of donors and oxygen as an acceptor (GO:0016641). The top 10 KEGG pathways were as follows: glycolysis/gluconeogenesis, the HIF-1 signaling pathway, central carbon metabolism in cancer, fructose and mannose metabolism, the PI3K-Akt signaling pathway, the Pentose phosphate pathway, proteoglycans in cancer, starch and sucrose metabolism, protein processing in the endoplasmic reticulum, and neomycin, kanamycin, and gentamicin biosynthesis (Figure 5).

### 3.5. Identification and Analysis Core Genes Coexpressed with P4HA1

**Figure 6:**
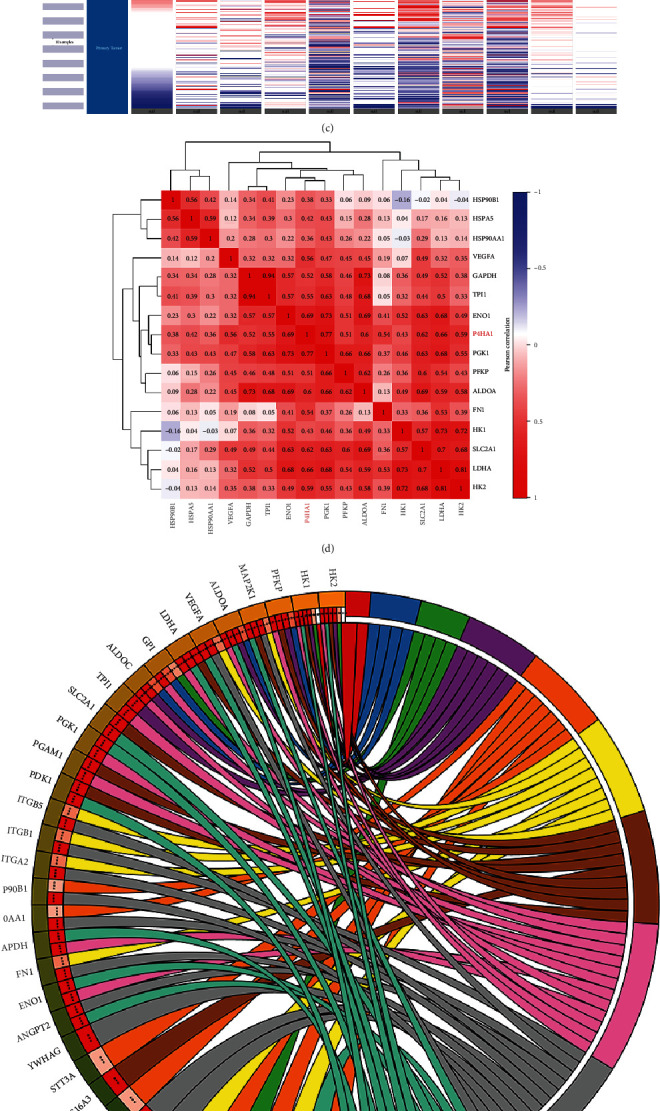
Construction of a protein-protein interaction (PPI) network of coexpressed genes of P4HA1 and analysis of Hub genes. (a) PPI network of coexpressed genes of P4HA1 was constructed and visualized using the STRING and Cytoscape tools, and the top 15 Hub genes were identified using CytoHubba tool kits in Cytoscape. (b) The most significant module in PPI networks was identified using MCODE tool kits in Cytoscape. (c) A heat map showing P4HA1 and top10 Hub genes expression in PAAD was constructed using UCSC. (d) Correlation clustering heat map of P4HA1 and top 15 Hub genes. (e) A circle diagram showing results of KEGG enrichment analysis of top 15 coexpressed genes.

A PPI network of the coexpressed genes was constructed using both the STRING database and Cytoscape software. Initially, the CytoHubba plug-in in the Cytoscape software was used to identify the top 15 node genes coexpressed with P4HA1 and were highlighted as potential key genes (Hub genes), based on the score of the degree among nodes. The genes included GAPDH, FN1, VEGFA, HSPA5, ENO1, PGK1, HSP90B1, TPI1, LDHA, HK1, HK2, ALDOA, PFKP, SLC2A1, and HSP90AA1 (Figure 6(a)). Afterwards, the MCODE plug-in was used to obtain the most important interaction module (Figure 6(b)). The results showed that Hub genes identified by the two methods were generally the same.

The expression heat map of P4HA1 and the Hub genes was displayed using the UCSC Cancer Genomics Browser. The findings showed that the expression pattern of 10 Hub genes was consistent with that of P4HA1 in pancreatic cancer (Figure 6C). Additionally, correlation heat maps showed that the expression of P4HA1 was positively associated with the expression patterns of these Hub genes (Figure 6(d)). Furthermore, the KEGG circus diagram of the top 15 Hub genes showed that they were mainly involved in the HIF-1 signaling pathway, the PI3K-Akt signaling pathway, glycolysis/gluconeogenesis, central carbon metabolism in cancer, proteoglycans in cancer, protein processing in the endoplasmic reticulum, fructose and mannose metabolism, and the pentose phosphate pathway(Figure 6(e)).

**Figure 7:**
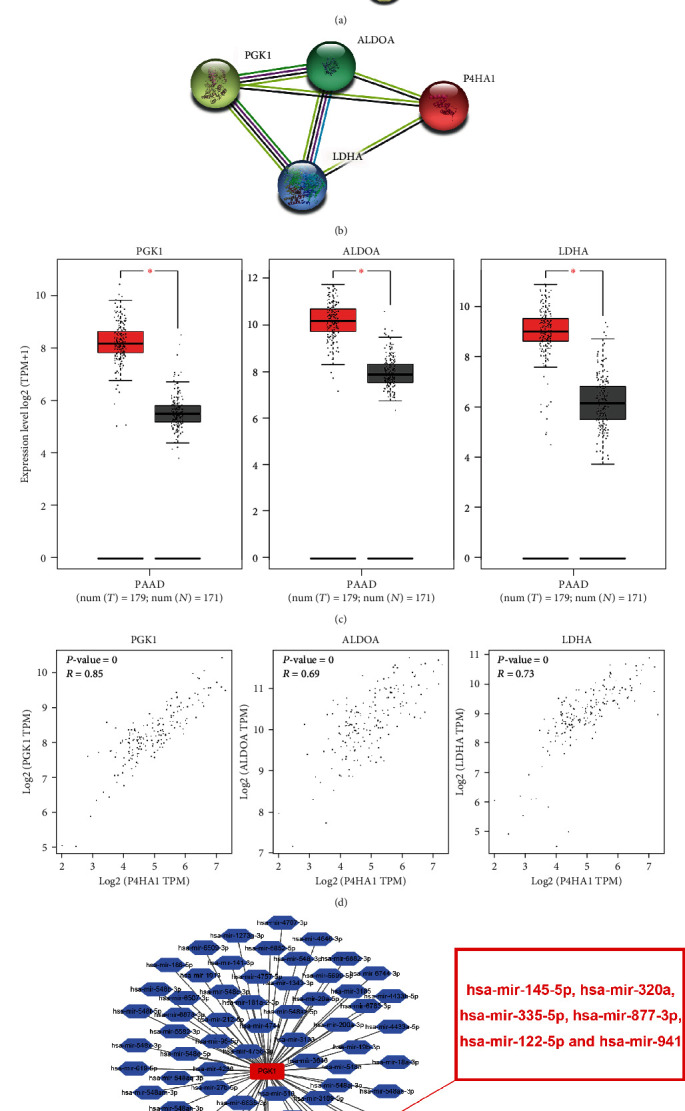
A PPI network showing Hub genes interacting with P4HA1 and the construction of a miRNA-mRNA network. (a) A PPI network showing the interaction among P4HA1 and its top 15 coexpressed genes; the interconnecting lines between those proteins indicate that there were interaction relationships between one and another. (b) A diagram of the PPI network for P4HA1 and three coexpressed genes directly interacting with P4HA1. (c) The expression of three coexpressed genes interacting with P4HA1 in PAAD using GEPIA. (d) Correlation diagram of the three coexpressed genes interacting with P4HA1 in PAAD using GEPIA. (e) The miRNA-mRNA network of the three coexpressed genes and P4HA1. Notably, hsa-miR-877-3p, hsa-miR-941, hsa-miR-320a, hsa-miR-324-5p, hsa-miR-122-5p, has-miR-145-5p, and hsa-miR-335-5p were predicted to target P4HA1.

Moreover, P4HA1 and the top 15 Hub genes were subjected to PPI analysis (Figure 7(a)). Hub genes that directly interacted with P4HA1 were selected, including PGK1, ALDOA, and LDHA (Figure 7(b)). Expression level and correlation analyses showed that these three genes were significantly overexpressed in pancreatic cancer (Figure 7(c)) and had a positive correlation with P4HA1 (Figure 7(d)). Therefore, the results suggested that PGK1, ALDOA, and LDHA may play important synergistic roles for P4HA1 in pancreatic cancer.

### 3.6. Prediction miRNA, lncRNA, and Building a ceRNA Regulatory Network

**Figure 8:**
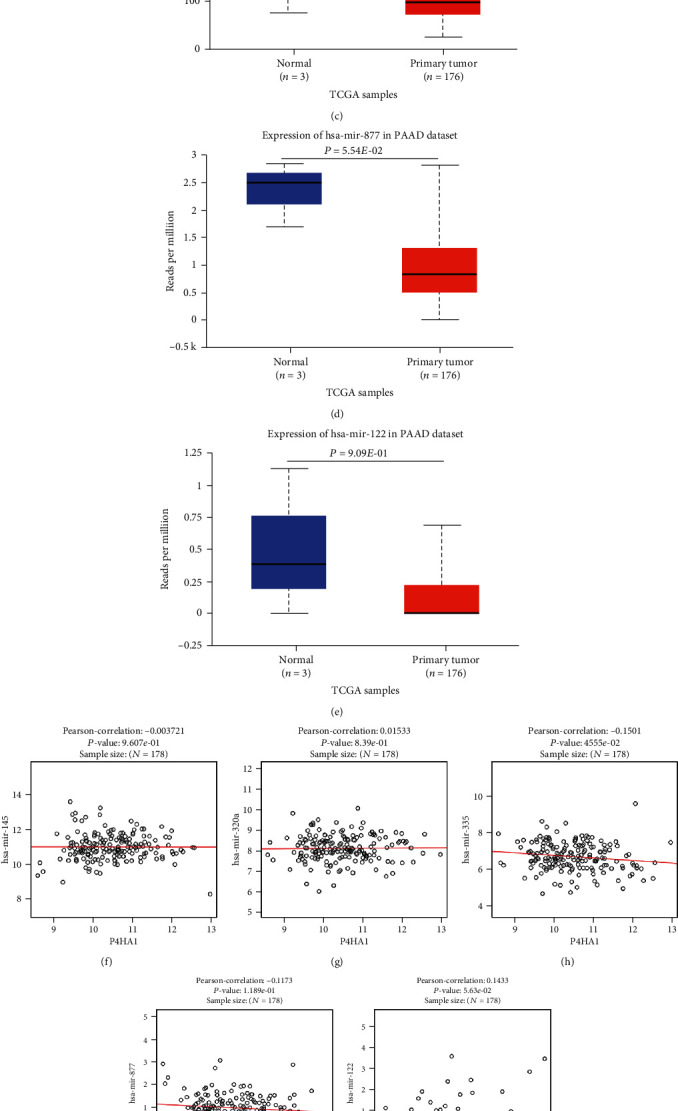
The expression, correlation, and prognostic value of 5 predicted upstream miRNAs of P4HA1. (a–e) The expression of 5 miRNAs in pancreatic cancer based on UALCAN. (f–j) Correlation between the expression of 5 miRNAs and P4HA1 expression in PAAD based on the UALCAN. (k) A forest map showing the prognostic value of 5 miRNAs in PAAD (TCGA). Green bars indicate a favorable prognosis; red bars indicate an unfavorable prognosis; black bars represent no significant statistical correlation. (l) The impact of miR-335-5p on overall survival of PAAD using Kaplan Meier-Plotter database.

**Figure 9:**
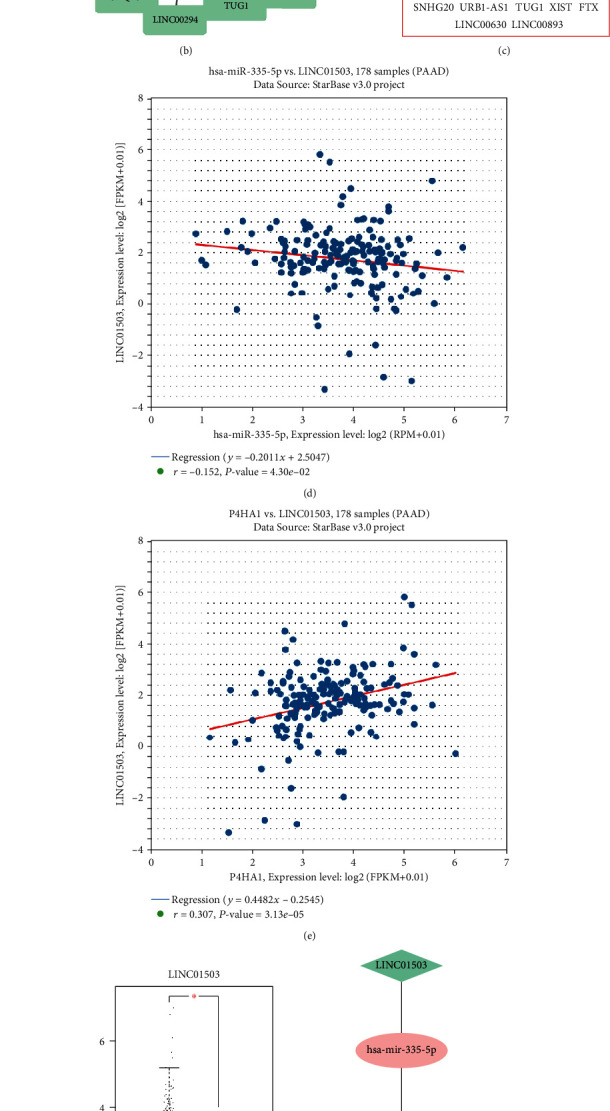
Identification of upstream lncRNAs of miR-335-5p, a ceRNA network, and a diagram showing a brief mechanism involving P4HA1 promote pancreatic cancer progress. (a) The predicted lncRNAs targeting miR-335-5p based on the StarBase database. (b) The potential lncRNAs targeting miR-335-5p as determined on the miRNet database. (c) 18 intersected lncRNAs obtained from the StarBase and miRNet databases. (d, e) Analysis of the correlation between LINC01503 and miR-335-5p and the correlation between LINC01503 and P4HA1. (f) LINC01503 expression in PAAD based on the analysis of GEPIA. (g) A ceRNA network was constructed. (h) A brief mechanism diagram was constructed.

In order to further explore the mechanism of P4HA1 in the prognosis of pancreatic cancer, the potential mechanism of noncoding RNA regulating P4HA1 was explored. First, NetworkAnalyst was used to predict the upstream miRNAs that could target and regulate P4HA1, including hsa-mir-145-5p, hsa-mir-320a, hsa-mir-335-5p, hsa-mir-877-3p, hsa-mir-122-5p, and hsa-mir-941 (Figure 8(e)). Afterwards, the UALCAN database, TCGA-based data, was used to analyze the expression of these miRNAs in pancreatic cancer (hsa-mir-941 was not reported in TCGA). The results showed that hsa-mir-320a and hsa-mir-335-5p had significantly low expression in pancreatic cancer (Figures 8(b) and 8(c)). Additionally, the LinkedOmics database was used to analyze the correlation between the five predicted miRNAs and P4HA1. The results showed that only miR-335-5p had a significant negative correlation with P4HA1 (Figure 8(h)). Furthermore, the Kaplan-Meier Plotter was used to analyze the effects of these miRNAs on the OS of patients with pancreatic cancer. The findings revealed that miR-122-5p was a poor prognostic marker [HR = 1.64, 95% CI (1.00-2.68), *P* = 0.046] while miR-335-5p was a protective factor in patients with pancreatic cancer [HR = 0.53, 95% CI (0.34-0.83), *P* = 0.0049]. However, the other three miRNAs had no effect on the overall survival of patients (Figures 8(k) and 9(l)). These results suggest that miR-335-5p was the most promising upstream miRNA regulating P4HA1.

Moreover, StarBase and miRNet were used to predict upstream lncRNA targeting to bind miR-335-5p. 31 lncRNAs were predicted from the StarBase database (Figure 9(a)) and 34 lncRNAs from the miRNet database (Figure 9(b)). 18 lncRNAs were predicted by both the two databases including SLFNL1-AS1, GAS5, MIR29B2CHG, SNHG8, TMEM161B-AS1, LINC01503, KCNQ1OT1, LINC00294, NEAT1, LINC00943, OIP5-AS1, SNHG20, URB1-AS1, TUG1, XIST, FTX, LINC00630, and LINC00893 (Figure 9(c)). The StarBase was then used to analyze the correlation between the predicted lncRNAs and miR-335-5p and the correlation between lncRNAs and P4HA1. As shown in Table 1, according to the regulatory characteristics of ceRNA theory, LINC01503 was screened out because it was negatively correlated with P4HA1 (*r* = 0.152, *P* = 4.30*E* − 02) (Figure 9(d)) and positively correlated with miR-335-5p (*r* = 0.307, *P* = 3.13*E* − 05) (Figure 9(e)). In addition, the expression of LINC01503 in PAAD was further verified on the GEPIA database. It was confirmed that LINC01503 was significantly overexpressed in PAAD (Figure 9(f)). These results suggested that LINC01503 was the most promising lncRNA targeting to regulate miR-335-5p.

Finally, based on the coexpressed genes, the miRNA and lncRNA selected above, a LINC01503/miR-335-5p/P4HA1 ceRNA regulatory axis was constructed (Figure 9(g)). Additionally, a brief model diagram involving the potential mechanism of P4HA1 underlying the progression of pancreatic cancer was drawn. (Figure 9(h)).

## 4. Discussion

P4HA1 is a key intracellular enzyme that catalyzes the formation of 4-hydroxyproline, and it is necessary for the correct three-dimensional folding of newly synthesized procollagen chains, thereby maintaining the steady state of the extracellular matrix^3^, ^29^. P4HA1 is involved in regulating extracellular collagen synthesis and secretion and regulating extracellular matrix components. However, increasing evidence shows that P4HA1 is related to the occurrence, invasion, and metastasis of some human cancers including breast cancer and prostate cancer, glioma, and hepatocellular carcinoma. Gilkes et al. 4 found that knocking down P4HA1 could reduce collagen deposition and inhibit the growth and metastasis of breast cancer cells. Additionally, the overexpression of P4HA1 was shown to promote the migration and metastasis of prostate cancer through the P4HA1-MMP1 signaling pathway^5^. Zhou et al. 7 found that the expression of P4HA1 may be regulated by HIF1*α*, and its overexpression could promote invasive growth of gliomas by upregulating EMT induced by SNAI1 and SNAI2 and by causing angiogenesis of HBMECs. It was also shown that P4HA1 could be targeted by miR-30e, thereby inhibiting cell proliferation and colony formation in hepatocellular carcinoma^30^. Therefore, previous studies showed that P4HA1 played an important role in the process of malignant tumor progression. However, it was unclear whether P4HA1 plays an important role in the occurrence and malignant progression of pancreatic cancer, which was worthy of further exploration.

In this study, a brief pan-cancer analysis of P4HA1 in various tumors was first conducted. This facilitated an understanding of the expression pattern and prognosis of P4HA1 in various tumors. In pancreatic cancer, P4HA1 not only was highly expressed but also was related to prognosis in patients. This provided a basis for further analysis of the relationship between P4HA1 and pancreatic cancer. The study then sought to further verify the correlation between expression of P4HA1 and prognosis of pancreatic cancer. To achieve this, GEO data from the Oncomine database was used to observe the expression level of P4HA1 in pancreatic cancer tissues compared to normal ones. It was shown that expression in pancreatic cancer was significantly higher than that in normal tissues, consistent with the results obtained from analysis using the GEPIA database. In addition, the HPA database confirmed that the level of the P4HA1 protein in pancreatic cancer was higher than that in normal pancreatic tissues. Moreover, this study found that high expression of P4HA1 was associated with poor clinicopathological features in pancreatic cancer. Our results showed that P4HA1 expression was not related to the M stage but considered the low number of M-positive patients included in the TCGA database; it may also have a certain deviation to our results. Therefore, this still needs to be confirmed by large-scale studies in the future. Subsequently, the prognostic analysis revealed that high expression of P4HA1 was related to poor prognosis in patients with pancreatic cancer. These results indicated that P4HA1 played an important and unfavorable role in the malignant transformation of pancreatic cancer and could be a promising biomarker of pancreatic cancer in the future.

In order to better understand the potential function of P4HA1 in pancreatic cancer, genes coexpressed with the P4HA1 were predicted. GO enrichment analysis (Figures 6(b)–6(d)) and KEGG pathway enrichment analysis (Figure 6(e)) of these genes showed that they were mainly involved in the biological processes of glycolysis, glucose metabolism, and production of ATP from ADP. The genes were mainly associated with glycolysis/gluconeogenesis, the HIF-1 signaling pathway, central carbon metabolism of cancer, fructose and mannose metabolism, the PI3K-Akt signaling pathway, and the pentose phosphate pathway. These results were consistent with previous reports on P4HA1 and collagen synthesis as well as tissue cell hypoxia^29^, ^31^, ^32^. Therefore, we speculated that P4HA1 plays an important role in tumors probably through these biological processes. Moreover, three genes (PGK1, ALDOA, and LDHA) directly interacting with P4HA1 were selected from the top 15 Hub genes. Analysis using the GEPIA database further confirmed that the genes were overexpressed in pancreatic cancer. Additionally, there was a significant positive correlation between the three genes and P4HA1. Therefore, the results suggested that these coexpressed genes might play an important synergistic role with P4HA1 in the progression of pancreatic cancer through glycolysis, HIF-1*α*, and metabolism.

Previous studies showed that the interaction of lncRNAs and miRNAs played an important role in the process of cancer and miRNAs could mediate tumor progression by regulating their downstream mRNA^12^. Therefore, in order to explore the potential regulatory mechanism of P4HA1 in the progression of pancreatic cancer, a lncRNA-miRNA-mRNA interaction network was constructed. First, six potential miRNAs were identified as candidates that targeted P4HA1 (miR-145-5p, miR-320a, miR-335-5p, miR-877-3p, miR-122-5p, and miR-941), and predicted targeting relationship was verified in TarBase and miRTarBase. Further, expression levels of the six potential miRNAs in pancreatic cancer were analyzed. The results showed that two of them (miR-320a and miR-335-5p) were significantly downregulated in pancreatic cancer. According to the ceRNA hypothesis, miRNA has a negative regulatory relationship with the target gene, so miR-335-5p was further screened with negative correlation between miRNA and P4HA1 by correlation analysis.

The effect of these miRNAs on the OS of patients with pancreatic cancer was then analyzed using the Kaplan-Meier Plotter database. The results further proved that miR-335-5p was a protective factor in pancreatic cancer [HR = 0.53, 95% CI (0.34-0.83), *P* = 0.0049]. Moreover, previous studies reported that miR-335-5p had tumor suppressive effects in lung cancer^33^, gastric cancer^34^, colorectal cancer^35^, and breast cancer^36^. Therefore, miR-335-5p was considered to be the most promising miRNA that could target and regulate P4HA1.

Similarly, StarBase and miRNet were used to predict upstream lncRNA targeting miR-335-5p. 18 lncRNAs predicted by the two both databases were obtained. According to the ceRNA theory, lncRNAs are negatively correlated with miRNAs but positively correlated with mRNA; therefore, the only one lncRNA (LINC01503) was eligible. The expression level of LINC01503 in pancreatic cancer was then examined and found that it was significantly upregulated in PAAD. In addition, previous studies identified LINC01503 played an oncogene role in tumors such as colorectal cancer^37^, gastric cancer^38^, glioma^39^, cholangiocarcinoma^40^, and cervical cancer^41^ and promotes tumor proliferation and invasion. Therefore, LINC01503 was considered to be the most promising lncRNA that could target and regulate miR-335-5p. Finally, the LINC01503/miR-335-5p/P4HA1 ceRNA regulatory axis was constructed by combining the miRNA and lncRNA selected in the above analytical process.

In summary, the study utilized bioinformatics tools to provide a reliable, comprehensive analysis of the expression pattern, prognostic value, and potential regulatory mechanism of P4HA1 in pancreatic cancer. It was confirmed that the overexpression of P4HA1 not only was associated with poor prognosis but was also related to poor clinicopathology in the disease. In addition, the potential regulatory mechanism of P4HA1 in promoting the progression of pancreatic cancer was explored by constructing a ceRNA regulatory axis.

## 5. Conclusion

P4HA1 can be used as a novel and promising prognostic biomarker for patients with pancreatic cancer, and it may provide a new therapeutic direction for the treatment of pancreatic cancer. However, considering that the findings were based on bioinformatics analyses, more laboratory experiments and clinical trials are needed to further validate in the future.

## Figures and Tables

**Table 1:** Correlation analysis between 18 lncRNAs and miR-335-5p and between 18 lncRNAs and P4HA1.

	miR-335-5p		P4HA1	
	Cor	*P* value	Cor	*P* value
SLFNL1-AS1	0.111	1.41*E* − 01	-0.140	6.28*E* − 02
GAS5	0.223	2.73**E** − 03	0.084	2.63*E* − 01
MIR29B2CHG	0.257	5.35**E** − 04	-0.135	7.14*E* − 02
SNHG8	0.451	2.56**E** − 10	-0.266	3.31**E** − 04
TMEM161B-AS1	0.277	1.85**E** − 04	-0.203	6.47**E** − 03
LINC01503	-0.152	4.30**E** − 02	0.307	3.13**E** − 05
KCNQ1OT1	-0.044	5.60*E* − 01	0.057	4.50*E* − 01
LINC00294	-0.146	5.23*E* − 02	-0.137	6.74*E* − 02
NEAT1	-0.033	6.65*E* − 01	0.190	1.09**E** − 02
LINC00943	-0.128	8.90*E* − 02	0.029	6.99*E* − 01
OIP5-AS1	-0.217	3.57**E** − 03	0.253	6.41**E** − 04
SNHG20	0.261	4.30**E** − 04	-0.294	6.73**E** − 05
URB1-AS1	0.200	7.33**E** − 03	-0.102	1.75*E* − 01
TUG1	-0.097	1.98*E* − 01	0.278	1.69**E** − 04
XIST	-0.031	6.84*E* − 01	0.057	4.51*E* − 01
FTX	0.056	4.56*E* − 01	0.127	9.24*E* − 02
LINC00630	-0.098	1.95*E* − 01	0.427	2.68**E** − 09
LINC00893	0.178	1.72**E** − 02	-0.066	3.84*E* − 01

Cor: *R* value of Pearson's correlation; bold values indicate *P* < 0.05.

## Data Availability

Source data of this study were derived from the public repositories, as indicated in Materials and Methods of the manuscript. And the datasets used and/or analyzed during the current study are available from the corresponding author on reasonable request.

## Abbreviations

P4HA1: Prolyl 4-hydroxylase subunit alpha 1
ceRNA: Competing endogenous RNA
lncRNA: Long noncoding RNA
MRE: miRNA recognition elements
ncRNA: Nonprotein coding RNA
KICH: Kidney Chromophobe
TCGA: The Cancer Genome Atlas
GTEx: Genotype-tissue expression
HPA: Human Protein Atlas
GO: Gene Ontology
KEGG: Kyoto Encyclopedia of Genes and Genomes
MOCDE: Molecular Complex Detection
PPI: Protein-protein interaction
CC: Cellular components
BP: Biological processes
MF: Molecular functions
FC: Fold change
OS: Overall survival
DSS: Disease-specific survival
PFI: Progress-free interval
DFI: Disease-free interval
DFS: Disease-free survival
HR: Hazard ratio
CI: Confidence intervals.
